# Enlargement of the Prostate: Its Treatment and Radical Cure

**Published:** 1895-03

**Authors:** 


*Enlargement of the Prostate: its Treatment and Radical Cure.* By C. W. Mansell Moullin, M.D. Pp. viii., 176. London: H. K. Lewis. 1894.

Since McGill of Leeds firmly advocated more radical measures in the treatment of enlargement of the prostate, the organ has been the subject of much study and writing, and the author of this work thinks the time has arrived for gathering together and recording what has been done in this field of surgery.

He has himself been a careful observer of prostatic disease, and the present volume forms an important addition to his writings on hypertrophy of the prostate. The task has been accomplished wisely and well, and the book may be taken as a trustworthy and comprehensive guide on this important disease. The view that the catheter is "almost the sole means for obtaining relief" is no longer tenable, and those who still think so will find much in these pages to interest and enlighten them. The book is clearly written and unusually free from typographical errors. The expression "supra pubes"—which we do not like, as it is neither English nor Latin—occurs on pp. 126, 148, and 163. The book is up to date, neatly bound and well printed, and contains an excellent bibliography and index.

